# Clonal evolution and blastic plasmacytoid dendritic cell neoplasm: malignancies of divergent hematopoietic lineages emerging from a common founding clone

**DOI:** 10.1038/s41375-024-02305-8

**Published:** 2024-06-18

**Authors:** Svenja Denker, Axel Künstner, Julian Schwarting, Hanno M. Witte, Veronica Bernard, Stephanie Stölting, Philipp Lohneis, Kathrin Kusch, Nikolas von Bubnoff, Hartmut Merz, Hauke Busch, Alfred C. Feller, Niklas Gebauer

**Affiliations:** 1 Medical Systems Biology Group, University of Lübeck, Ratzeburger Allee 160, 23538 Lübeck, Germany; 2 University Cancer Center Schleswig-Holstein, University Hospital of Schleswig-Holstein, Campus Lübeck, 23538 Lübeck, Germany; 3 Department of Hematology and Oncology, University Hospital of Schleswig-Holstein, Campus Lübeck, Ratzeburger Allee 160, 23538 Lübeck, Germany; 4 Hämatopathologie Lübeck, Consultation Centre for Lymph Node Pathology and Hematopathology, 23562 Lübeck, Germany; 5 Department of Hematology and Oncology, Federal Armed Forces Hospital Ulm, Oberer Eselsberg 40, 89081 Ulm, Germany

**Keywords:** Leukaemia, Cancer genomics

## To the Editor:

Blastic plasmacytoid dendritic cell neoplasm (BPDCN) is a rare and aggressive blood cancer that originates from clonal pDCs or their precursors. A shared clonal origin between BPDCN and syn- and metachronous myeloid neoplasms, is assumed in up to 20% of cases [1]. Recurrently observed clonal pDCs in chronic myelomonocytic leukemia (CMML) and clonal hematopoiesis (CH) detection in approximately 65% of BPDCN cases underscore this concept [2]. Differential diagnosis between BPDCN and neighboring entities including acute myeloid leukemia (AML) with pDC-like features is challenging. In our recent study, published in LEUKEMIA, we employed epigenetic profiling to complement conventional diagnostics and uncover molecular mechanisms determined by DNA-methylation in BPDCN [3]. Throughout this study, we encountered several cases of metachronous non-BPDCN hematological malignancies both before and after BPDCN diagnosis and while one aim of the said study was, to differentiate BPDCN from its neighboring entities, we observed the sequential development of BPDCN and several of these malignancies (including CMML, AML and even a peripheral T-cell lymphoma) in a subset of patients, suggestive of a common clonal origin as reported previously [2].

Here we present two unique BPDCN cases where sequential whole exome sequencing (WES) delineated clonal evolution from an initial presentation of lymphoid cancers (T-lymphoblastic lymphoma (T-LBL) and ALK-negative anaplastic large-cell lymphoma (ALCL)) and clonally related CH in the bone marrow to BPDCN and in one case AML. This represents the first comprehensive investigation of clonal evolution in BPDCN patients diagnosed with metachronous lymphoid malignancies.

**Fig. 1:**
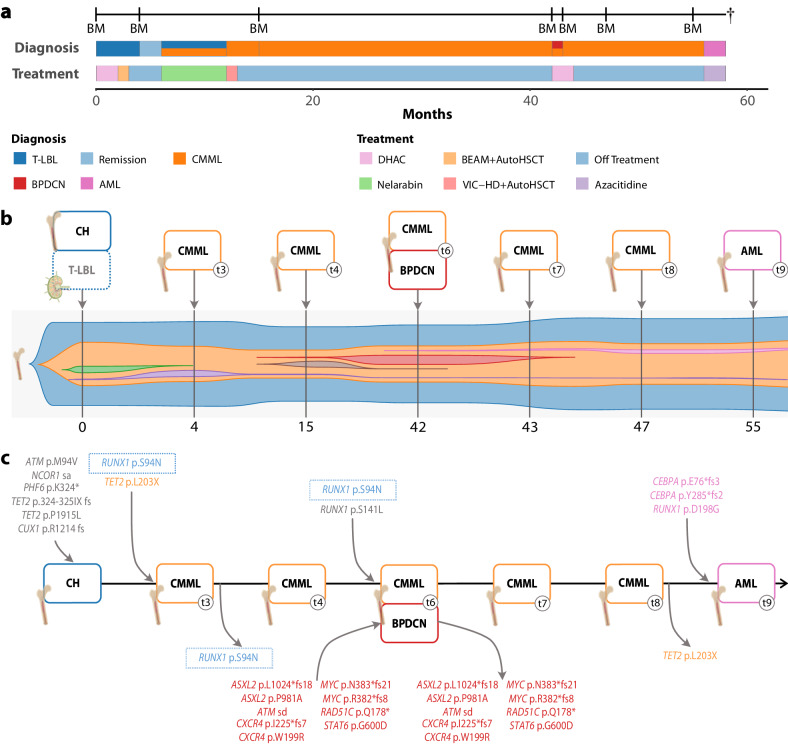
Clinical and molecular characteristics of Case 1. **a** Accurate temporal delimitation of diagnosis, treatment, and follow-up. **b** Clonal evolution and composition of sequential hematological malignancies originating from a shared CH origin, represented non-proportionally over time (T-LBL was not successfully sequenced). **c** Sequential acquisition and/or loss of mutations affecting pathogenetically relevant genes (manual curation was performed based on pre-described pathogenetic implications in the respective entities) across all sequenced samples including tissue origin of sequenced biopsies; dotted boxes represent variants recurrently gained and/or lost.

**Fig. 2:**
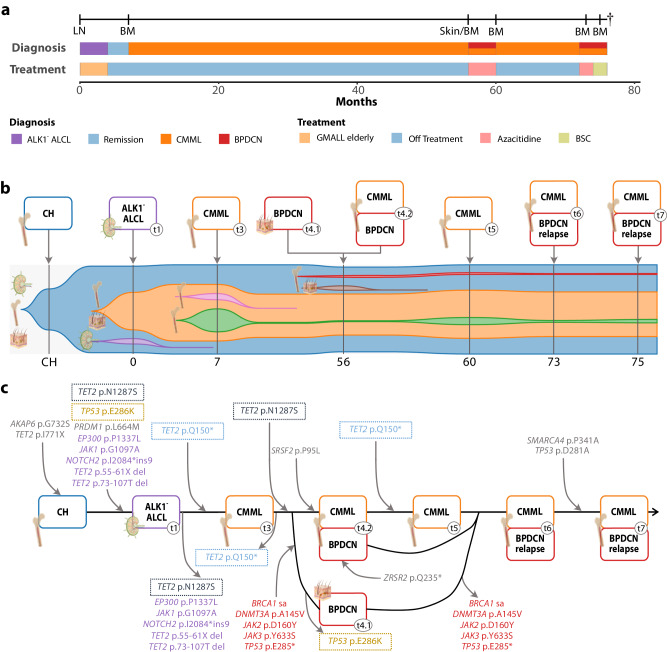
Clinical and molecular characteristics of Case 2. **a** Accurate temporal delimitation of diagnosis, treatment, and follow-up. **b** Clonal evolution and composition of sequential hematological malignancies originating from a shared CH origin, represented non-proportionally over time. Spatial annotation of clones is provided. **c** Sequential acquisition and/or loss of mutations affecting pathogenetically relevant genes (manually curated) across all sequenced samples including tissue origin of sequenced biopsies; dotted boxes represent variants recurrently gained and/or lost.

Clinical and molecular case presentations are provided in Figs. 1 and 2, the Supplementary Materials and Methods, and Supplementary Table 1. The molecular and bioinformatics workflow was performed as described and is summarized in the Supplementary Materials and Methods [4]. All somatic variants detected throughout the study are summarized in Supplementary Table 2 regarding the respective patient and time point.

For Case 1 two somatic *TET2* mutations (324-325IX fs and P1915L) persist in all samples except the germline, at a mean Variant Allele Frequency (VAF) of 35% (range 11–58%). Library preparation from the initial T-LBL for WES failed. The chronologically first sequenced sample represents the baseline staging bone marrow at T-LBL diagnosis which already revealed CH. Bone marrow at remission after first and second autologous stem-cell transplantation (ASCT) revealed a clonally evolved malignancy, with CMML features that acquired a (subclonal) *RUNX1* mutation alongside an additional *TET2* mutation. After over two years of surveillance, the patient developed BPDCN accompanied by mutations in *ASXL2*, *ATM*, *CXCR4*, *MYC*, *RAD51C* and *STAT6*. Hematopathological examination ruled out (pDC-like) AML according to current WHO and ICC criteria, for which *RUNX1* mutations constitute typical mutational drivers [5]. Following successful treatment, bone marrow biopsies in remission revealed persistent CMML from which a second aggressive transformation developed, undergoing a lineage shift into AML which was morphologically and immunophenotypically divergent from the previous BPDCN manifestation (now MPO^+^, CD33^+^, and CD34^+^), accompanied by the acquisition *RUNX1* and *CEBPA* (p.Y285*fs2 and p.E76*fs3) mutations which rapidly progressed while the patient received best supportive care due to his impaired performance status [5]. 22 genomic aberrations were identified across all samples, indicative of a common founding clone originating from clonal hematopoiesis with subsequent dissemination across CMML, BPDCN, and ultimately AML. The founding clone alongside its mutational profile was maintained throughout both ASCTs. No graft samples were available to confirm the preservation and subsequent evolution of the founding clone molecularly [6]. Phylogenomics revealed a markedly branched tree with a significant divergence between the early manifestations of CMML compared to both BPDCN as well as later CMML and AML samples (Supplementary Fig. 2A). It is tempting to speculate regarding a causal relationship between CH and radiotherapy for breast cancer on two occasions throughout the patient’s history.

In Case 2, we detected 50 somatic variants across all samples. A somatic *TET2* I771X mutation persisted throughout all samples (VAF mean 36%, range 7–54%), corresponding to a founding event for all subsequent malignancies stemming from a shared hematopoietic clone. Originating from a preexisting CH clone the patient primarily developed ALK^-^ ALCL with typical additional mutations, including previously reported mutational drivers of ALCL (*EP300*, *JAK1, NOTCH2*, and *PRDM1*) [7]. Chemotherapy according to an age-adjusted protocol induced remission. However, a subsequent bone marrow biopsy revealed a clonally evolved CMML with an additionally acquired *TET2* Q150*. Over four years later, the patient developed cutaneous BPDCN manifestations. Simultaneously, bone marrow trephine biopsy revealed the preexisting CMML with minimal BPDCN infiltrates (5–10% cellularity). Mutations in *DNMT3A*, *JAK2*, *TP53*, and *ZRSR2* marked clonal evolution from CMML to BPDCN. Following successful treatment with azacytidine, CMML persistence in the bone marrow was observed followed by subsequent treatment-refractory BPDCN relapse which additionally developed mutations in *SMARCA4* and *TP53*. Subsequently, the patient received best supportive care and succumbed to rapidly progressive BPDCN. Phylogenomic analysis revealed markedly different mutational drivers resembling different evolutionary paths between ALCL and early CMML compared to later CMML and BPDCN, indicating early clonal divergence yet a shared origin (Supplementary Fig. 2B).

Utilizing over-representation analysis against HALLMARK gene sets and oncogenic signatures we observed enrichment in mutations affecting UV response in BPDCN, as described, alongside a pattern affecting the mitotic spindle apparatus (p-values 0.00029 and <0.00001 respectively, data not shown) [8, 9].

Sequencing was performed on bulk samples, gathered before the approval of tagraxofusp. Sorted cell populations or single-cell sequencing including patients treated with novel immune therapies could have provided even deeper insights, particularly in samples concurrently exhibiting CMML and BPDCN infiltrates. We significantly expand the molecular understanding of the potential impact of CH on the clinical course of BPDCN patients, through the first description not only of the metachronous emergence of T-cell neoplasia in BPDCN patients but also the exhaustive molecular analysis of the intricate clonal relationship and evolution of these malignancies originating from a shared CH clone that is preserved through ASCT and sequentially crosses linage boundaries [2, 10]. Adding to our previous study, we do not only depict molecular divergences but also similarities and potential evolutionary trajectories in BPDCN patients.

### Supplementary information

Supplementary Materials and Methods

Supplementary Table 02

## Data Availability

BAM files from WES have been deposited in the European genome-phenome archive (EGA) under the accession numbers EGAS00001006166 and EGAS50000000214.
