# Supplementary material for: Clonal evolution and blastic plasmacytoid dendritic cell neoplasm: malignancies of divergent hematopoietic lineages emerging from a common founding clone
Source: Leukemia. 2024 Jun 18;38(8):1858–61. doi: 10.1038/s41375-024-02305-8 (PMC11286505; doi:10.1038/s41375-024-02305-8)
Supplement: Supplementary file 1 — Supplementary Materials and Methods [file 41375_2024_2305_MOESM1_ESM.docx]

**Supplementary Materials and Methods**

# Clinical case presentations:

**Case 1:**

A 69-year-old female patient with pleural and pericardial effusion, causing dyspnea, exhibited mediastinal bulk, mesenteric lymphadenopathy, and a malignant lesion in the left kidney, leading to a diagnosis of T-LBL. Previously, she had undergone curative surgery, adjuvant radiotherapy, and anti-hormonal treatment for breast cancer 16 years prior, with a similar tumor in the contralateral breast nine years later, treated similarly. Initially the patient received DHAC (carboplatin, cytarabine, dexamethasone), high-dose BEAM (carmustine, cytarabine, etoposide, melphalan) with autologous stem cell transplantation (ASCT). Relapse at six months was salvaged with nelarabine, high-dose VIC (etoposide, ifosfamide, carboplatin), and a second ASCT, inducing sustained remission. Bone marrow assessments revealed low-risk CMML-0 with a significant pDC (CD123^+^, CD4^+^, CD56-) component. After 30 months of surveillance, progression to BPDCN (CD123^+^, CD4^+^, CD56^+^, TCL1^+/-^) occurred, which was managed with DHAC. BPDCN comprised 45% of marrow cellularity at diagnosis, while the remainder exhibited CMML infiltrates and residual hematopoiesis. The BPDCN component responded for twelve months before relapse as acute myeloid leukemia (AML) (partially CD34^+^, partially CD117^+^, partially CD56^+^, MPO^+^). Intensive treatment was no longer clinically feasible and azacytidine was initiated but failed to yield a meaningful response. The patient deceased 56 months post-T-LBL diagnosis.

**Case 2:**

A 71-year-old male presented with ALK-negative anaplastic large cell lymphoma (ALCL; stage IIIB). Polychemotherapy induced a complete response. Bone marrow biopsies at diagnosis and remission revealed clonal hematopoiesis (CH) and subsequently CMML-0. After 49 months of surveillance, rapid onset cutaneous BPDCN occurred, alongside minor bone marrow infiltrates (approximately 10% cellularity), with remaining CMML and an associated pDC population (CD123^+^, CD4^+^, CD56^+/-^). Azacytidine achieved a complete response in BPDCN, while CMML persisted. Due to compromised performance status (ECOG 2-3) and patient preference, treatment was suspended, resulting in BPDCN relapse after twelve months. Re-initiation of azacytidine failed to yield a response, and the patient died 74 months post-ALCL diagnosis.

# Whole exome sequencing (WES)

For genomic characterization, FFPE samples were collected from lymphoid tumors at diagnosis as well as from bone marrow and skin at various time points, and for Case 1 colorectal mucosa. The study was approved by the ethics committee of the University of Lübeck (reference-no 18-311) and conducted following the declaration of Helsinki. All samples underwent WES on an Illumina NovaSeq instrument, following library preparation using Agilent SureSelect Human All Exon V6 library preparation kit (Agilent Technologies) from regions with high (> 50%) tumor cell content, ensured when required, by microdissection, as described^16^. Regrettably, library preparation failed repeatedly for the T-LBL of Case 1 and the germline control of Case 2.

# Exome Data Processing and Variant Calling

Raw sequencing data (in fastq format) was used as input for the nfcore workflow (nextflow v23.04.2) sarek (v3.2.3^17^) to perform variant calling against GRCh38 as reference genome. Briefly, reads were trimmed (adapter and quality values) by applying fastp (v0.23.4^18^) and mapping was performed using bwa mem2 (v2.2.1^19^). Afterward, mate-pair information was fixed, PCR duplicates were removed, and base quality recalibration was performed using Picard Tools^20^, GATK (v4.4.0.0^21^) and dbSNP v146^22^. Next, variant calling was performed in tumor-only mode (Mutect2^23^) with gnomAD (r2.1.1^24^) as a resource of known germline variants. Early bone marrow samples as putative germline samples were tested for the presence of clonal hematopoiesis (CH) mutations; all tested samples carried CH mutations ^25^. Therefore, variant calling was performed in tumor-only mode throughout the study. Variants were left aligned (GATK LeftAlignAndTrimVariants) and only variants with a PASS filter flag were kept for variant annotation using Variant Effect Predictor (VEP v103, GRCh38^26^; adding CADD v1.6^27^, dbNSFP v4.1a, and gnomAD r3.0^28^ as additional annotations) ^29^.

The obtained annotated variants were filtered according to the following criteria: variants present in the 1000g panel of normal were removed, and only variants with population frequencies of ≤ 0.001 in gnomAD were considered. Variants outside coding regions and variants that were not deletions, insertions, or SNPs were also removed. The retained variants were required to have a minimum variant allele frequency of 1%, a minimum alternative allele coverage of 5, and a minimum coverage of 30.

Genes classified as tumor suppressors or oncogenes by Vogelstein et al.^30^, those associated with BPDCN^31^, CMML^32^, oncoKB actionable genes, and CHIP genes identified by Niroula et al. were selected as candidate genes. To be considered, the following criteria were applied: a CADD score greater than 10, minimum coverage of 20, a minimum alternative allele coverage of 4, or a variant allele frequency greater than 10%. Variants found in the germline were further examined to ensure a minimum variant allele frequency of 35%.

For variants identified as described above, the presence at additional time points was checked in the unfiltered variant set and, if present, they were rescued and manually curated; germline variants were filtered with a variant allele frequency of > 35 % to obtain the final dataset.

**Copy Number Aberrations and Loss of Heterozygosity Estimation**

Copy number aberrations (CNAs) were identified using Control-FREEC (v11.6^33^) in tumor-only mode as implemented in the sarek. For each sample, CNA calls were converted to BED format, and CNA ratios were calculated for overlapping regions; resulting median ratios were *log_2_* transformed (*log_2_(ratio)-1*). The *log_2_* transformed ratios were used as copy number estimations to infer clonal architecture (described below).

Regions with signs of loss of heterozygosity (LOH) were identified using cnv_facets (v0.16^34^) which is based on FACETS^35^ with the putative germline as normal control. Additionally, the dbSNP (v151, GRCh38) database was used as source of common, polymorphic sites.

# Phylogeny and clonal architecture

The phylogeny of each case's samples was reconstructed using Treeomics (v1.9.2^36^). The following settings were used: a sequencing error rate *e* of 1%, a prior absent probability **c_0_* of 50%, a max absent variant allele frequency of 1%, a false discovery rate of 5%, a false-positive rate of 0.5%, and an absent classification minimum coverage of 100. The WES filtering parameter was set to true, and the candidate genes described above were used as driver genes. For each case, the LOH frequency was calculated by dividing the sum of the LOH lengths per sample by the genome length of 3.3 × 10^9^, followed by the calculation of the median. False-positive variants identified by Treeomics were excluded from any further interpretation.

The subclonal architecture of the cases was determined using SciClone (v1.1.1^37^) by analysing its samples except for the germline sample. Copy number aberrations from Control-FREEC were utilized as copy number calls while LOH regions (identified by cnv_facets) were excluded. Additionally, the sex chromosomes were not considered in the analysis.

The results were analyzed using ClonEvol (v0.99.11^38^) to infer the clonal evolution. The analysis was performed using the infer.clonal.models method with a bootstrap subclonal test with a non-parametric model. The following parameters were also used: cluster.center set to mean, minimum cluster vaf of 0.001, cancer initiation model set to polyclonal, sum.p set to 0.05, and alpha set to 0.05. Figures 1 and 2 were partially created with biorender.com.

# Statistical Analysis

Unless specified otherwise, statistical analysis was conducted using R (v4.3.2), and the following R packages were utilized for analysis and visualization: Tidyverse (v2.0.0^39^) for data management; maftools (v2.18.0^40^) for summarizing, analyzing, and visualizing variant data; and VCFR (v1.14.0) for extracting LOH information from the vcf INFO field. Visualizing the clonal evolution was performed using TimeScape (v1.26.0^41^) by utilizing the output of the ClonEvol (v0.99.11^38^) analysis results and calculating the mean variant allele frequency of the cluster centers.

Over-representation analysis was conducted against HALLMARK gene sets (MSigDB v2023.2.Hs) and oncogenic gene sets by applying a hypergeometric test (hypeR v2.1.0); gene sets with p < 0.01 were considered significant ^42-44^.

# Supplementary Figure legends

**Supplementary Figure 1.** Histopathological characteristics of Case 1 (**a-g**) and Case 2 (**h-n**) captured employing a Keyence BZ-X800 microscope.

(**a**) Bone marrow with CH constellation exhibiting suspicious features including disturbed architecture (ASDCL) and enrichment in monocytes (CD14) and megakaryocytes (CD42) but not yet diagnostic of MDS/MPN or CMML. (**b, c**) CMML (ASDCL, CD14) with an expanded population of pDCs (CD123). (**d**) Bone marrow showing concurrent infiltrates of CMML and BPDCN, clearly distinguishable by marker expression of subpopulations arranged in patterns (ASDCL, CD56, and CD123). (**e, f**) Bone marrow controls showing BPDCN remission with persistence of CMML after therapy (ASDCL, CD14, CD56, CD123). (**g**) Secondary acceleration in AML, morphologically and immunophenotypically distinct from BPDCN (blasts: CD34^+^, CD56^+^ only in a subpopulation and MPO^+^). Images were captured using a 20x objective.

(**h**) ALK^-^ ALCL with typical morphology (H&E, Giemsa) and strong positivity for CD30. (**i**) CMML (ASDCL, CD14) with dysplasia with the megakaryopoiesis. (**j**) Subcutaneous infiltrates with typical morphology (Giemsa) and immunophenotype of BPDCN (CD56, CD123). (**k**) Bone marrow with concomitant infiltrates of the pre-existing CMML (ASDCL) and minor infiltrates of BPDCN (CD56 and CD123). (**l**) Remission after BPDCN treatment resulted in CMML (ASDCL) persistence without indications for residual BPDCN infiltrates (CD56, CD123). (**m, n**) BPDCN relapse (CD56, CD123) re-emerging in front of a CMML background. Morphologically inconspicuous bone marrow with CH constellation at ALCL diagnosis not shown. Images were captured using 20x and 40x (Giemsa in 2a and 2c, large CD56 in 2f) objectives respectively**.**

**Supplementary Figure 2.** Phylogenetic tree inferred by Treeomics of Case 1 (**a**) and Case 2 (**b**)

# Supplementary Tables

**Supplementary Table 1.** Baseline clinicopathologic characteristics.

| **Characteristics** | **Case 1** | **Case 2** |
| --- | --- | --- |
| **Age at BPDCN diagnosis** | 75 | 76 |
| **Sex** | f | m |
| **Syn-/metachronous hematologic neoplasm (clonally related)** | (T-LBL*), CMML, AML | ALK-negative ALCL, CMML |
| **Syn-/metachronous solid tumors** | Breast cancer left sided (16 yrs prior to 1^st^ hematologocal diagnosis)  Breast cancer right sided (9 yrs prior to 1^st^ hematologocal diagnosis) | - |
| **BPDCN manifestation**   - No. involved sites - No. extranodal sites - Skin - Bone marrow | 6  3  No  Yes | 3  2  Yes  Yes |
| ECOG-PS | 1 | 1 |
| LDH (ref. normal level 240 U/l) | 3000 | 339 |
| **Immunohistochemistry**  **(initial BPDCN diagnosis)**  BPDCN specific   - CD56^+^ - CD123^+^ - TCL1^+^   Immature lineage markers   - TdT^+^ - CD34^+^   B-lineage markers   - CD79a^+^   T-lineage markers   - CD3^+^ - CD4^+^ - CD8^+^   Myeloid lineage markers   - CD33^+^ - CD117^+^ - MPO^+^   Ki-67 | +  +  +/-  -  -  +  -  +  +/-  +/-  -  -  80 % | +  +  +  +/-  -  +/-  +/-  +  -  +  -  -  50% |
| ALCL, anaplastic large cell lymphoma; ALK, anaplastic lymphoma kinase; AML, acute myeloid leukemia; CMML, chronic myelomonocytic leukemia; ECOG, Eastern Cooperative Oncology Group; LDH, lactate dehydrogenase; T-LBL, T-lymphoblastic lymphoma.  Legend:  + > 60% of surface area  +/- 10 – 60% of surface area  - < 10% of surface area  * Clonal relation could not be evaluated due to failed library preparation upon repeated attempts. | | |

**Supplementary Table 2.** Identified variants after filtering.

*Please see separate file*

**Supplementary References**

16. Witte HM, Kunstner A, Hertel N, Bernd HW, Bernard V, Stolting S*, et al.* Integrative genomic and transcriptomic analysis in plasmablastic lymphoma identifies disruption of key regulatory pathways. *Blood Adv* 2021 Oct 29.

17. Garcia M, Juhos S, Larsson M, Olason PI, Martin M, Eisfeldt J*, et al.* Sarek: A portable workflow for whole-genome sequencing analysis of germline and somatic variants. *F1000Res* 2020; **9:** 63.

18. Chen S, Zhou Y, Chen Y, Gu J. fastp: an ultra-fast all-in-one FASTQ preprocessor. *Bioinformatics* 2018 Sep 1; **34**(17)**:** i884-i890.

19. M. Vasimuddin SM, H. Li and S. Aluru. Efficient Architecture-Aware Acceleration of BWA-MEM for Multicore Systems. 2019 IEEE International Parallel and Distributed Processing Symposium (IPDPS), Rio de Janeiro, Brazil; 2019.

20. Institute B. Picard Toolkit. 2019 [cited; Available from: <http://broadinstitute.github.io/picard/>

21. McKenna A, Hanna M, Banks E, Sivachenko A, Cibulskis K, Kernytsky A*, et al.* The Genome Analysis Toolkit: a MapReduce framework for analyzing next-generation DNA sequencing data. *Genome Res* 2010 Sep; **20**(9)**:** 1297-1303.

22. Sherry ST, Ward MH, Kholodov M, Baker J, Phan L, Smigielski EM*, et al.* dbSNP: the NCBI database of genetic variation. *Nucleic Acids Res* 2001 Jan 1; **29**(1)**:** 308-311.

23. Cibulskis K, Lawrence MS, Carter SL, Sivachenko A, Jaffe D, Sougnez C*, et al.* Sensitive detection of somatic point mutations in impure and heterogeneous cancer samples. *Nat Biotechnol* 2013 Mar; **31**(3)**:** 213-219.

24. Karczewski KJ, Francioli LC, Tiao G, Cummings BB, Alfoldi J, Wang Q*, et al.* The mutational constraint spectrum quantified from variation in 141,456 humans. *Nature* 2020 May; **581**(7809)**:** 434-443.

25. Niroula A, Sekar A, Murakami MA, Trinder M, Agrawal M, Wong WJ*, et al.* Distinction of lymphoid and myeloid clonal hematopoiesis. *Nat Med* 2021 Nov; **27**(11)**:** 1921-1927.

26. McLaren W, Gil L, Hunt SE, Riat HS, Ritchie GR, Thormann A*, et al.* The Ensembl Variant Effect Predictor. *Genome Biol* 2016 Jun 6; **17**(1)**:** 122.

27. Rentzsch P, Witten D, Cooper GM, Shendure J, Kircher M. CADD: predicting the deleteriousness of variants throughout the human genome. *Nucleic Acids Res* 2019 Jan 8; **47**(D1)**:** D886-D894.

28. Chen S, Francioli LC, Goodrich JK, Collins RL, Kanai M, Wang Q*, et al.* A genomic mutational constraint map using variation in 76,156 human genomes. *Nature* 2024 Jan; **625**(7993)**:** 92-100.

29. Liu X, Li C, Mou C, Dong Y, Tu Y. dbNSFP v4: a comprehensive database of transcript-specific functional predictions and annotations for human nonsynonymous and splice-site SNVs. *Genome Med* 2020 Dec 2; **12**(1)**:** 103.

30. Vogelstein B, Papadopoulos N, Velculescu VE, Zhou S, Diaz LA, Jr., Kinzler KW. Cancer genome landscapes. *Science* 2013 Mar 29; **339**(6127)**:** 1546-1558.

31. Künstner A, Schwarting J, Witte HM, Bernard V, Stölting S, Kusch K*, et al.* Integrative molecular profiling identifies two molecularly and clinically distinct subtypes of blastic plasmacytoid dendritic cell neoplasm. *Blood Cancer Journal* 2022 2022/07/04; **12**(7)**:** 101.

32. Tyner JW, Tognon CE, Bottomly D, Wilmot B, Kurtz SE, Savage SL*, et al.* Functional genomic landscape of acute myeloid leukaemia. *Nature* 2018 Oct; **562**(7728)**:** 526-531.

33. Boeva V, Popova T, Bleakley K, Chiche P, Cappo J, Schleiermacher G*, et al.* Control-FREEC: a tool for assessing copy number and allelic content using next-generation sequencing data. *Bioinformatics* 2012 Feb 1; **28**(3)**:** 423-425.

34. CNV_FACETS [cited; Available from: <https://github.com/dariober/cnv_facets?tab=readme-ov-file>

35. Shen R, Seshan VE. FACETS: allele-specific copy number and clonal heterogeneity analysis tool for high-throughput DNA sequencing. *Nucleic Acids Res* 2016 Sep 19; **44**(16)**:** e131.

36. Reiter JG, Makohon-Moore AP, Gerold JM, Bozic I, Chatterjee K, Iacobuzio-Donahue CA*, et al.* Reconstructing metastatic seeding patterns of human cancers. *Nat Commun* 2017 Jan 31; **8:** 14114.

37. Miller CA, White BS, Dees ND, Griffith M, Welch JS, Griffith OL*, et al.* SciClone: inferring clonal architecture and tracking the spatial and temporal patterns of tumor evolution. *PLoS Comput Biol* 2014 Aug; **10**(8)**:** e1003665.

38. Dang HX, White BS, Foltz SM, Miller CA, Luo J, Fields RC*, et al.* ClonEvol: clonal ordering and visualization in cancer sequencing. *Ann Oncol* 2017 Dec 1; **28**(12)**:** 3076-3082.

39. Wickham H, Averick M, Bryan J, Chang W, D’Agostino L, Francois R*, et al.* Welcome to the Tidyverse. *The Journal of Open Source Software* 2019; **4**(43).

40. Mayakonda A, Lin DC, Assenov Y, Plass C, Koeffler HP. Maftools: efficient and comprehensive analysis of somatic variants in cancer. *Genome Res* 2018 Nov; **28**(11)**:** 1747-1756.

41. Smith M. timescape: Patient Clonal Timescapes. . 2023 [cited; Available from: <https://bioconductor.org/packages/timescape>.

42. Liberzon A, Birger C, Thorvaldsdottir H, Ghandi M, Mesirov JP, Tamayo P. The Molecular Signatures Database (MSigDB) hallmark gene set collection. *Cell Syst* 2015 Dec 23; **1**(6)**:** 417-425.

43. Sanchez-Vega F, Mina M, Armenia J, Chatila WK, Luna A, La KC*, et al.* Oncogenic Signaling Pathways in The Cancer Genome Atlas. *Cell* 2018 Apr 5; **173**(2)**:** 321-337 e310.

44. Federico A, Monti S. hypeR: an R package for geneset enrichment workflows. *Bioinformatics* 2020 Feb 15; **36**(4)**:** 1307-1308.
